# State-Space Algorithms for Estimating Spike Rate Functions

**DOI:** 10.1155/2010/426539

**Published:** 2009-11-05

**Authors:** Anne C. Smith, Joao D. Scalon, Sylvia Wirth, Marianna Yanike, Wendy A. Suzuki, Emery N. Brown

**Affiliations:** ^1^Department of Anesthesiology and Pain Medicine, One Shields Avenue, TB-170, UC Davis, Davis, CA 95616, USA; ^2^Departamento de Ciências Exatas, Universidade Federal de Lavras, 37200-000 MG, Brazil; ^3^Centre de Neuroscience Cognitive, CNRS, 69675 Bron, France; ^4^Department of Neuroscience, Columbia University, New York, NY 10032, USA; ^5^Neuroscience Statistics Research Laboratory, Department of Anesthesia and Critical Care, Massachusetts General Hospital/Harvard Medical School, Boston, MA 02114, USA; ^6^Division of Health Sciences and Technology, Harvard Medical School/Massachusetts Institute of Technology, Cambridge, MA 02139, USA

## Abstract

The accurate characterization of spike firing rates including the determination of when changes in activity occur is a fundamental issue in the analysis of neurophysiological data. Here we describe a state-space model for estimating the spike rate function that provides a maximum likelihood estimate of the spike rate, model goodness-of-fit assessments, as well as confidence intervals for the spike rate function and any other associated quantities of interest. Using simulated spike data, we first compare the performance of the state-space approach with that of Bayesian adaptive regression splines (BARS) and a simple cubic spline smoothing algorithm. We show that the state-space model is computationally efficient and comparable with other spline approaches. Our results suggest both a theoretically sound and practical approach for estimating spike rate functions that is applicable to a wide range of neurophysiological data.

## 1. Introduction

When does a neuron respond to an external sensory stimulus or to a motor movement? When is its maximum response to that stimulus? Does that response change over time with experience? Neurophysiologists and statisticians have been trying to develop approaches to address these questions ever since this experimental approach was developed. One of the most widely used approaches used to determine when and if a neuron fired to the stimulus is to use a peristimulus time histogram (PSTH), simply averaging the responses over some time bin over all the trials collected. However, because there is no principled way of choosing the bin size for the PSTH, its interpretation is difficult. An even more challenging question is characterizing neural activity of responses to a stimulus if it changes over time as is the case in learning. Again, averaging techniques are typically used to characterize changes across trials, but averaging across 5 or 10 trials severely limits the temporal resolution of this kind of analysis. Beyond averaging techniques, a range of more sophisticated statistical methods have been applied to characterize neural activity including regression or reverse correlation techniques [1], maximum likelihood fitting of parametric statistical models [2–9], and Bayesian approaches [10–13].

Recently models have been proposed for the analysis of spike train data using the state-space approach [4, 14, 15]. The state-space model is a standard approach in engineering, statistics, and computer science for analyzing dynamic hidden or unobservable processes [15–18, 23]. It is defined by two equations: the state equation that defines the evolution of the hidden or implicit stimulus through time and the observation equation that links the implicit stimulus to the neural response. Analysis using simulated neural spike train data established the feasibility and accuracy of this state-space approach [15]. We previously used a point process adaptive filter in the analysis of a study in which learning-related neural activity was characterized in the hippocampus as monkeys learned new associations online [19, 20]. This filter algorithm provided highly accurate spike rate functions that allowed analysis of the neural activity both within a trial and across learning trials. Using these algorithms we identified changes in neural activity that were correlated with behavioral learning over the course of the training session. However, because confidence intervals were not calculated for this first model, it did not allow us to define statistically when within or across trials, a change in firing rate took place.

To address this issue, we now describe a state-space model for estimating the spike rate function by maximum likelihood using an approximate Expectation-Maximization (EM) algorithm. A major advance of this model over our previous model is that we can now assess model goodness-of-fit and compute confidence intervals for the spike rate function and other associated quantities of interest such as location of maximal firing. In this way, one can determine the precise timing of neural change either within or across trials. Using simulated spike rate data, we first compare our approach with that of Bayesian adaptive regression splines (BARS, [13, 21]) and a simple cubic spline smoothing algorithm. The state-space model performs comparably with BARS (in its default setting) and improves over the cubic spline method. Next, we illustrate the state-space algorithm applied to real neurophysiological data from the monkey hippocampus during the performance of an associative learning task [20]. To test the model on a wide range of neural data, we also apply the state-space algorithm to real spike counts from the supplementary eye field of a macaque monkey during saccadic eye movements analyzed in 10-millisecond bins [22]. We show that this modified state-space algorithm provides both an accurate and highly flexible way to describe spike rate functions over a wide range of experiments.

## 2. Materials and Methods

### 2.1. A State-Space Model of Neural Spiking Activity

We assume that the spike rate function of a single neuron is a dynamic process that can be studied with the state-space framework used in engineering, statistics, and computer science [15–18, 23]. The state-space model consists of two equations: a state equation and an observation equation. The state equation defines an unobservable state process that governs the shape of the spike rate function across time. Such state models with unobservable processes are often [15, 24, 25] referred to as hidden Markov or latent process models. The observation equation completes the state-space model setup and defines how the observed data relate to the unobservable state process. The data we observe in the neurophysiological experiments are the series of spike trains. Therefore, the objective of the analysis is to estimate the state process and hence, the spike rate function from the observed data. We conduct our analysis of the experiment from the perspective of an ideal observer. That is, we estimate the spike rate function at each time point having recorded the entire spike train or set of spike trains.

Assume that during a neurophysiological experiment in which the spiking activity of a single neuron is recorded for *J* trials and that each trial is of length *T*. For an experiment involving a single neural spike train we have *J* = 1. We define the observation interval (0, *T*] and the conditional intensity function for *t* ∈ (0, *T*] as

(1) $$\lambda \left(t\;|\;H_{t}\right)=\underset{\Delta \to 0}{\lim\;}\;\frac{\text{Pr}\left(N\left(t+\Delta \right)-N\left(t\right)=1\;|\;H_{t}\right)}{\Delta },$$

where *N*(*t*) is the number of spikes in the interval (0, *t*] and *H*_*t*_ is the history up to time *t*. The conditional intensity function is a history-dependent rate function that generalizes the definition of the Poisson rate [26]. If the point process is an inhomogeneous Poisson process, then the conditional intensity function is *λ*(*t* ∣ *H*_*t*_) = *λ*(*t*). It follows that *λ*(*t* ∣ *H*_*t*_)Δ is the probability of a spike in [*t*, *t* + Δ) when there is history dependence in the spike train. In survival analysis the conditional intensity is termed the hazard function because, in this case, *λ*(*t* ∣ *H*_*t*_)Δ measures the probability of a failure or death in [*t*, *t* + Δ) given that the process has survived up to time *t* [27].

To facilitate presentation of the model, we divide the time period (0, *T*] into *K* intervals of equal width Δ = *TK* ^−1^, so that there is at most one spike per interval. Let *n*_*jk*_ be the number of spikes in the interval ((*k* − 1)Δ, *k*Δ] for trial *j*, where, *j* = 1,…, *J* and *k* = 1,…, *K*. We define the state model as

(2) $$x_{k}=x_{k-1}+\varepsilon_{k},$$

where *x*_*k*_ is the unknown state at time *k*Δ and *ε*_*k*_ is a Gaussian random variable with mean zero and variance *σ*_*ε*_^2^. We assume further that *x*_0_ is Gaussian with mean *μ* and variance *σ*_0_^2^.

Using the theory of point processes [26, 28], we express the observation model for the spikes *n*_*jk*_ in the interval ((*k* − 1)Δ, *k*Δ] given *x*_*k*_ as

(3) $$\text{Pr}\left(n_{jk}\right)=\exp\;\left\{n_{jk}\;\log\;\lambda \left(k\Delta \;|\;x_{k}\right)\Delta -\lambda \left(k\Delta \;|\;x_{k}\right)\Delta \right\},$$

where we model the conditional intensity function in terms of the state process as

(4) $$\lambda \left(k\Delta \;|\;x_{k}\right)=\exp\;\left(x_{k}\right).$$

Under this model, the spiking activity on different trials is independent and history dependence in the spiking activity within a trial is defined in terms of the state process. We use the exponential function to ensure that the right hand side in (3) is strictly positive.

We define *n*_*k*_ = (*n*_1*k*_,…, *n*_*Jk*_) as all the observations in the interval ((*k* − 1)Δ, *k*Δ] across all *J* trials, *N*_1:*K*_ = (*n*_1_,…, *n*_*K*_), *x* = (*x*_1_,…, *x*_*K*_), and *θ* = (*μ*, *σ*_*ε*_^2^). Because *x* is unobservable and *θ* is an unknown parameter, we use the Expectation-Maximization (EM) algorithm to compute their estimates by maximum likelihood [29]. The EM algorithm is a well-known procedure for performing maximum likelihood estimation when there is an unobservable process or missing observations. We used the EM algorithm to estimate state-space models from point process observations with linear Gaussian state processes [15]. Our EM algorithm is a special case of the EM algorithm in Smith and Brown [15], and its derivation is given in Appendix A. We denote the maximum likelihood estimate of *θ* as $\hat{\theta }=\left(\hat{\mu },{\hat{\sigma }}_{\varepsilon }^{2}\right)$.

To understand what is accomplished in the EM model fitting, we note that the log of the joint probability density of the spike train data and the state process (A.1) is

(5) $$\overset{J}{\underset{j=1}{\sum }}\left[\overset{K}{\underset{k=1}{\sum }}\left(\left(n_{jk}\;\log\;\lambda \left(k\Delta \;|\;x_{k}\right)\Delta \right)-\left(\lambda \left(k\Delta \;|\;x_{k}\right)\Delta \right)\right)\right]-{\left(2\sigma_{\varepsilon }^{2}\right)}^{-1}\overset{K}{\underset{k=2}{\sum }}{\left(x_{k}-x_{k-1}\right)}^{2}.$$

Expression 2.5 has the form of a penalized likelihood function and shows that the values of the state process impose a stochastic smoothness constraint on the conditional intensity or spike rate function [18, 24]. The parameter *σ*_*ε*_^2^ is the smoothing parameter. The larger the value of *σ*_*ε*_^2^, the rougher the estimate of the spike rate function or the PSTH. Similarly, the smaller the value of *σ*_*ε*_^2^, the smoother the estimates of these functions. Hence, the maximum likelihood estimate of *σ*_*ε*_^2^ governs smoothness of the spike rate function or PSTH. That is, the analysis uses maximum likelihood to estimate the degree of smoothing that is most consistent with the data.

### 2.2. Estimating the Spike Rate Function

Given the maximum likelihood estimates of the *x* and *θ*, we can compute for each *x*_*k*_, *x*_*k*∣*K*_, the smoothing algorithm estimate of the state process at time *k*Δ. It is the estimate of *x*_*k*_ given *N*_1:*K*_, all the data in the experiment with the parameter *θ* replaced by its maximum likelihood estimate, where the notation *x*_*k*∣*K*_ means the learning state process estimate at trial *k* given the data up through trial *K*. The smoothing algorithm gives the ideal observer estimate of the state process. The smoothing algorithm estimate of the state process at each time *k*Δ is the Gaussian random variable with mean *x*_*k*∣*K*_ and variance, *σ*_*k*∣*K*_^2^. The conditional intensity function is computed by (4) evaluated at the maximum likelihood estimates of *x*_*k*_ and *θ* and is defined as

(6) $$\lambda \left(k\Delta \;|\;x_{k\;|\;K}\right)=\exp\;\left(x_{k\;|\;K}\right)$$

for *k* = 1,…, *K*.

### 2.3. Confidence Intervals for the Spike Rate Function

Approximating the probability density of the state at *k*Δ as the Gaussian density with mean *x*_*k*∣*K*_ and variance *σ*_*k*∣*K*_^2^, it follows from (6) and the standard change of variable formula from probability theory [30] that the probability density of the spike rate function at time *k*Δ is the lognormal probability density defined as [15]

(7) $$p\left(\lambda_{k}\;|\;x_{k|K},\hat{\theta }\right)={\left(2\pi \sigma_{\varepsilon }^{2}\right)}^{-1/2}{\lambda_{k}}^{-1}\exp\;\left\{-{\left(2\sigma_{\varepsilon }^{2}\right)}^{-1}{\left(\log\;\lambda_{k}-x_{k\;|\;K}\right)}^{2}\right\},$$

where *λ*_*k*_ = *λ*(*k*Δ ∣ *x*_*k*∣*K*_). A standard analysis is to construct a histogram from the data collected across the *J* trials in the experiment. Under the state-space model, we can compute the probability density of a histogram constructed with any bin width. To see this, we note that given two times 0 ≤ *t*_1_ ≤ *t*_2_ ≤ *T* the smoothed histogram based on our conditional intensity function estimate is

(8) $$\Lambda \left(t_{2}-t_{1}\right)=\int_{t_{1}}^{t_{2}}\lambda \left(u\right)du,$$

and hence, the smoothed rate function estimate is

(9) $$\hat{\Lambda }\left(t_{2}-t_{1}\right)=\int_{t_{1}}^{t_{2}}\hat{\lambda }\left(u\right)du\approx \overset{}{\underset{t_{1}\leq k\Delta \leq t_{1}}{\sum }}\lambda \left(k\Delta \;|\;x_{k\;|\;K},\hat{\theta }\right)\Delta .$$

The confidence intervals for the smoothed estimate of the rate function in (9) can be efficiently computed by Monte Carlo methods. The details of these computations are given in Appendix B.

### 2.4. Between Time Comparisons for the Spike Rate Function

An objective of the spike rate function or PSTH analysis is to compare rate functions between two or more time points in the observation interval (0, *T*]. That is, for any two times *k*_1_Δ and *k*_2_Δ, we can compute Pr(*λ*_*k*_2_Δ_ > *λ*_*k*_1_Δ_). As in Smith et al. [31] we compute this probability using Monte Carlo methods. The details of this computation are given in Appendix C.

### 2.5. Model Assessment

An important part of our analysis is to assess how well the model estimates the true function in the presence of noise. To determine this, we designed a simulation study to test our estimation method across a range of rate curves with differing noise levels. We compared the estimated function and true function using the average mean squared error (MSE). For our assessments of goodness-of-fit in the real data cases, we used the chi-squared test. This tests the extent to which the observed number of spikes in a prespecified time interval is consistent with the numbers predicted by the model [32].

### 2.6. Alternative Methods for Estimating Spike Rate Functions

We compare our state-space smoothing methods to two established procedures for data smoothing: cubic splines and Bayesian adaptive regression splines.

#### 2.6.1. Cubic Splines

Cubic splines are a standard method for smoothing of both continuous-valued and discrete data [24]. They are composed of third-order polynomials that are continuous up to order three and differentiable up to order two. Given a specification of the knot locations, they provide a smooth estimate of the underlying function.

#### 2.6.2. Bayesian Adaptive Regression Splines

Bayesian adaptive regression splines (BARS) is a recently developed procedure for smoothing both continuous-valued and discrete data [12, 21, 33]. The method assumes that the underlying rate function can be described by a set of free-knot cubic B-splines. BARS uses the Bayesian information criterion (BIC) in conjunction with variable dimension Markov chain Monte Carlo methods to estimate the spline coefficients, to estimate the location and number of knots and to decide on the order of the B-splines used in the analysis. The mode of the corresponding marginal posterior probability density is taken as the estimate of each quantity. BARS has been shown to outperform other spline-based smoothing procedures (e.g., [34]) in terms of mean squared error [21].

### 2.7. Experimental Protocol for a Location Scene-Association Task

To illustrate the performance of our methods in the analysis of an actual learning experiment, we analyze the responses of neural activity in a macaque monkey performing a location-scene association task, described in detail in Wirth et al. [20]. The objective of the study was to relate the animal's behavioral learning curve to the activity of individually isolated hippocampal neurons [20]. In this task, the monkey fixates on a point on a computer screen for 300 milliseconds and is then presented with a novel scene for 500 milliseconds. A delay period of 700 milliseconds follows, and in order to receive a reward, the monkey has to associate the scene with the correct one of four target locations: north, south, east, and west. Once the delay period ends, the monkey indicates its choice by making a saccadic eye movement to the chosen location. Typically between 2–4 novel scenes were learned simultaneously and trials of novel scenes are interspersed with trials in which four well-learned scenes are presented. Because there are four locations the monkey can choose as a response, the probability of a correct response occurring by chance is 0.25.

### 2.8. Experimental Protocol for a Study of Supplemental Eye Field Activity

As a second illustration of our methods we consider spike data recorded from the supplementary eye field (SEF) of a macaque monkey [22]. Neurons in the SEF play a role in oculomotor processes. A standard paradigm for studying the spiking properties of these neurons is a delayed eye movement task. In this task, the monkey fixates, is shown locations of potential target sites, and is then cued to the specific target to which it must saccade. Next, a preparatory cue is given, followed a random time later by a go signal. Upon receiving the go signal, the animal must saccade to the specific target and hold fixation for a specified amount of time in order to receive a reward. Beginning from the point of the specific target cue, neural activity is recorded for a fixed interval of time beyond the presentation of the go signal. After a brief rest period, the trial is repeated. Multiple trials from an experiment such as this are jointly analyzed using a PSTH to estimate firing rate for a finite interval following a fixed initiation point. That is, the trials are time aligned with respect to a fixed initial point, such as the target cue. The data across trials are binned in time intervals of a fixed length, and the rate in each bin is estimated as the average number of spikes in the fixed time interval.

## 3. Results

### 3.1. Simulation Study

**Figure 1:**
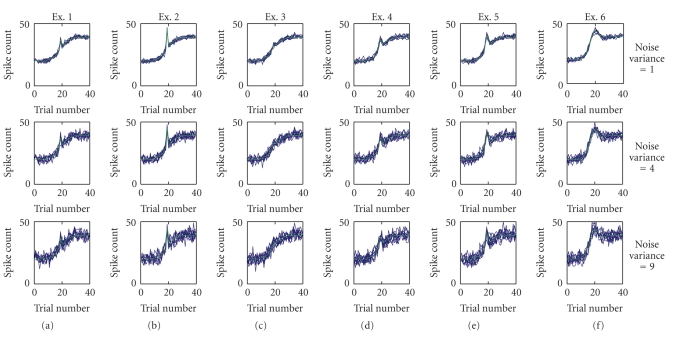
Test curves for simulation study. The six true functions (denoted Examples 1–6, green curves) are generated using a sigmoid combined with a Gaussian (10) using the parameter pairs for height of Gaussian, *H*, and width of Gaussian, *s*, of (10, .5), (20, .5), (10, 1), (20, 1), (30, 1), and (100, 3). Each row shows 10 noisy test sets superimposed (blue) and generated by adding zero-mean normally-distributed random noise to the true functions. The values of noise variance are 1 (top row), 4 (middle row) and 9 (bottom row).

We first designed a simulation study to compare our state-space smoothing method with BARS and splines. This study tests the ability to reproduce accurately test curves in the presence of noise. We constructed a true function of the form

(10) $$N_{k}=N_{0}+\frac{\left(N_{K}-N_{0}\right)}{1+\exp\;\left(-\gamma \left(k-\delta \right)\right)}+\frac{H}{\sqrt{2\pi s^{2}}}\exp\;\left(-\frac{{\left(k-\delta \right)}^{2}}{2s^{2}}\right)$$

for *k* = 1,…, *K. * This is a sigmoid-shaped curve with a small Gaussian increase close to the inflection point. Our choices for start point (*N*_0_), end point (*N*_*K*_), inflection point (*δ*), and the rate of increase of the sigmoid (*γ*) were, respectively, 20, 40, 20, and 0.3. We considered 6 combinations of the pair of parameters *H* and *s,* namely, (10, .5), (20, .5), (10, 1), (20, 1), (30, 1), and (100, 3), denoted Examples 1–6, respectively, (green curves in Figure 1). With these parameters, the maximum deviation resulting from the Gaussian (i.e., maximum of the last term in (10)) ranges from approximately 4 (Example 3) to approximately 16 (Example 2).

To simulate count data, we added to each of the 6 test curves zero-mean, Gaussian noise with a variance of either *σ*_*ν*_^2^ = 1, 4, or 9, and we rounded the continuous-valued observations to the nearest integer. For each noise variance, we drew 10 samples, resulting in a 6 × 3 × 10 test curves (blue curves in Figure 1). By using this choice of test parameters, we were able to compare how well the three methods reconstruct the true curves with very small deviations (Examples 1 and 3), very sudden changes (Examples 2 and 5) and broader deviations (Examples 4 and 6), all at three different noise levels (rows 1–3 in Figure 1). We chose this approach for the test curves because we determined empirically that it produced count data similar to those in the experiments of Wirth et al. [20] as well as rate functions similar in shape to the curves used to test BARS ([21, Example 2, Figure 1(b)]). The values used for noise variance were selected to range from sufficiently high that in some cases the Gaussian stimulus is barely perceptible (e.g., Example 3 with *σ*_*ν*_^2^ = 9) to relatively low such that the stimulus dominates (e.g., Example 2 with *σ*_*ν*_^2^ = 1) with signal to noise ratios (*sd*(*N*_1:*K*_)/*σ*_*ν*_) ranging from approximately 3 to 9.

For this study, we compared our state-space model estimates with those of BARS and splines using the mean squared error computed from

(11) $$\text{MSE}=\frac{1}{K}\overset{K}{\underset{k=1}{\sum }}{\left({\hat{N}}_{k}-\mathrm{int}\;\left(N_{k}\right)\right)}^{2},$$

where ${\hat{N}}_{k}$ is the count estimate computed from each of the three methods and int(*N*_*k*_) is computed from (10).

We considered two formulations for our state-space model. For the first naïve model (SS1), we estimated the initial rate at *k* = 0 from the first three data observations. For the second model (SS2), we reversed the data and estimated the end point (which is the true start point) by maximum likelihood. We then used this maximum likelihood estimate of the initial conditon at *k* = 1 as a fixed initial condition in model SS2. This takes advantage of the fact that a stationary time series taken forward in time should also apply with time reversed [35]. In practical terms, by adding more certainty in the SS2 model, the resulting random walk variance is often smaller resulting in smoother results.

**Figure 2:**
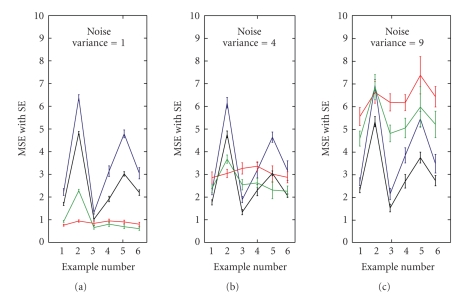
Average mean squared errors (MSEs) computed for the simulation study. We show results for SS1 (red), SS2 (black), BARS (blue) and splines (green) for Examples 1–6 at three different noise levels. With SS1 and splines the MSE increases as the noise level increases whereas SS2 and BARS give more consistent results across the range of noise levels.

For the lowest noise case (*σ*_*ν*_^2^ = 1), we found that SS1 and spline estimates had the lowest average MSEs of all the methods (red and green lines, resp., Figure 2). For the SS1 model this MSE was relatively constant across all 6 Examples. The spline model was also relatively constant except in Example 2 where there was a larger MSE and a very sudden change in the true function. For this low noise case, the SS2 estimates (black) were slightly better than BARS (blue) for all examples, though not as good as the SS1 and spline estimates. The MSEs from both BARS and SS2 were particularly high for Examples 2 and 5 where the change in true function was quite sudden at the inflection point and for Examples 4 and 6 where there was a broader bump. For Example 3, where the Gaussian bump is barely perceptible, all four methods were comparable.

**Figure 3:**
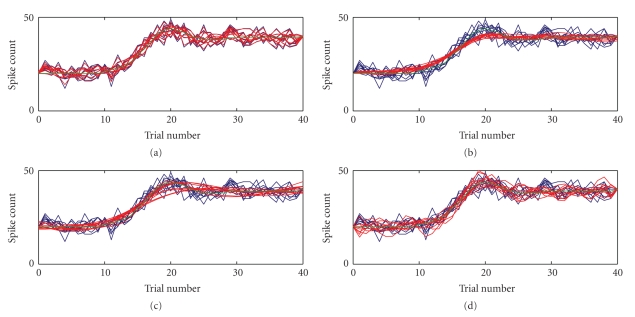
Example of performance of all four techniques applied to data from Example 6 with noise variance of 3. We show SS1 (a), SS2 (b), BARS (c), and splines (d). On each figure we show the raw count data (blue), mean estimated count (red), and true function used to generate the data (green). Each panel shows the 10 raw data curves and 10 estimated counts superposed. The SS1 and splines methods tend to track the noise whereas SS2 and BARS have more smoothing.

 As the noise variance increases to *σ*_*ν*_^2^ = 4 and 9, SS2 estimates had significantly lower MSEs (Figure 2) with the exception of the splines model in just one of the twelve parameter combinations (*σ*_*ν*_^2^ = 4 in Example 5). The SS2 MSE estimates were similar in trend to those of BARS though slightly lower. Again the cases where the true function changes suddenly are least well reproduced. The MSEs for SS1 are flat across all examples but become progressively higher in value as *σ*_*ν*_^2^ increases. This is because SS1 tends to track the noise in the count data without smoothing as we show for the high noise case of Example 6 (red lines, Figure 3(a)). As with the SS1 method, the spline estimates (Figure 3(d)) also appear to track the noise in the process, resulting in a ragged estimate of spike count. In contrast, SS1 and BARS estimates are smoother (red lines, Figures 3(b) and 3(c), resp.), but at the cost of smoothing out the Gaussian bump in the true curve (green).

Because SS1 appears to track the noise in the data without sufficient smoothing, we use only the SS2 approach for the following cases applied to real data.

**Data Example 1: comparing the changes in firing rate within trials in an location-scene association experiment**

**Figure 4:**
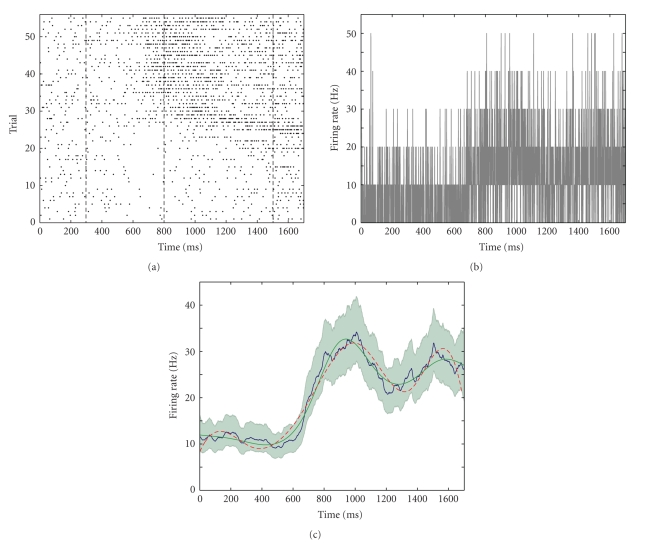
(a) Raster plot of raw spike data for a single cell over 55 trials. The four behavioral periods (baseline, scene, delay, and response) are delineated by the vertical dashed lines. (b) Peristimulus time histogram (PSTH) for the data with bin size of 1 millisecond. (c) Firing rates computed by state-space model (blue), BARS (green), and splines (red). The 95% confidence bounds for the state-space model are shaded in gray.

As a first illustration of our method applied to real data, we take the data from one hippocampal cell from the macaque monkey performing the location-scene association task described in Section 2. The data consists of spike times recorded at 1 millisecond precision from 55 repeated trials (Figure 4(a)). The average firing rate across the experiment was 20.42 Hz. We can see from the spike raster that the density of spikes increases both within trials and across trials.

One current strategy for estimating changes in firing rate as a function of time from the start of each trial is to employ the peristimulus time histogram (PSTH). The PSTH sums the observed spikes across trials and displays them as a histogram-type plot of counts occurring within fixed intervals of time. The choice of time interval or bin width is often made somewhat arbitrarily by the experimenter based on the desired degree of smoothing.

First, we applied our state-space algorithm to the count data summed (Figure 4(b)) at the precision of the experiment. The mean firing rate (blue curve, Figure 4(c)) yields similar firing rate estimates as the histogram but with the addition of a 95% confidence region (gray). For comparison we also computed the firing rate estimates using BARS (red dashed) and splines with 100 evenly spaced knots (green). All models give more interpretable results than the raw data (Figure 4(b)) as it is binned on such a small time scale that it is very noisy. The cubic splines method estimates the firing rate to be lower than observed at both ends.

To assess how well each model fits the data we carried out the *χ* ^2^ goodness-of-fit test. The null hypothesis here is that the model fits the data. We found that the results from both the SS (*χ* ^2^ = 1.57 × 103, *P* = .98) and BARS (*χ* ^2^ = 1.62 × 103, *P* = .88) models were consistent with this hypothesis and fit the data well. The splines approach had a low probability of fitting the data (*P* < .001).

**Figure 5:**
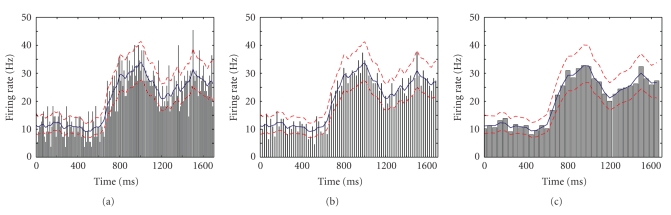
State-space approach applied to data from previous figure binned at different time precisions. We show data bin widths of 10 (a), 20 (b), and 50 (c). The estimated mean firing rate (blue curves) tends to be smoother as the bin width increases. The 95% confidence bounds (red dashed curves) remain relatively constant in width for all three cases.

To examine the effects of choice of bin width on the analysis of this data, we resorted the raw data into bins with widths of 10 milliseconds (gray bars, Figure 5(a)), 20 milliseconds (gray bars, Figure 5(b)), and 50 milliseconds (gray bars, Figure 5(c)). As the bin width increases, the histogram becomes smoother. We found that the SS estimates of instantaneous firing rate (blue lines, Figure 5) track all the PSTHs well.

A major advantage of the SS approach over the other options is that it provides confidence bounds (red dashed curves, Figure 5) and allows smoothing that captures the essential features of the firing rate curve for different bin widths without rerunning the computer code. That is, once we have the SS estimates at the finest precision, say 1 millisecond, it is straightforward and fast to get estimates of the firing rates for count spikes occurring within any fixed intervals of time using (9). Splines and BARS require a new run of the estimation procedure for every change in bin width. In addition, the SS method requires the estimation of only two parameters to get the firing rate curve while BARS requires six parameters to estimate the curve. For the splines estimates we required 100 internal evenly spaced knots to fit the curve.

**Figure 6:**
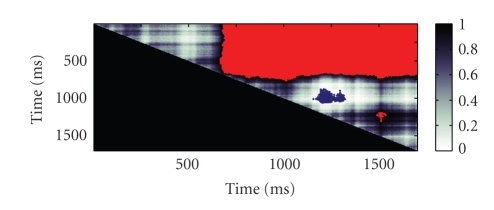
Trial-by-trial comparison between firing rates shown in Figure 4(c). Each pixel represents the value of the probability that the firing rate at time *i* (*x*-axis) is greater than the firing rate at time *j* (*y*-axis). The probability values are represented using the grayscale shown. Pixels with values greater that 0.99 are shown in red and pixels with values less than 0.01 are shown in blue. Therefore from approximately 650 milliseconds onwards the firing rate is significantly greater than previous firing rates (red region). The firing rate around 1250 milliseconds is lower than firing rates centered at 1000 milliseconds (blue region). For a small period around 1500 milliseconds the firgin rate is greater than the firing rate around 1250 milliseconds.

An important question here is whether the instantaneous firing rate is significantly different across the 1700-millisecond length of the experiment. Using the Monte Carlo algorithm presented in Appendix C, we are able to compute Pr(*i* > *j*), the probability that firing rate at time *i* was greater that the firing rate at time *j* for all *i* < *j* (Figure 6). By using this algorithm, it is possible to observe from the data that the following hold. The instantaneous firing rate observed in the first 634 milliseconds of the trial (baseline period and part of the scene presentation) was significantly smaller than the firing rates later than 634 milliseconds. The firing rate around 1250 milliseconds is lower than at times around 1000 milliseconds. The firing rate around 1500 milliseconds is significantly above the rate around 1250 milliseconds and the rate before 750 milliseconds.

**Figure 7:**
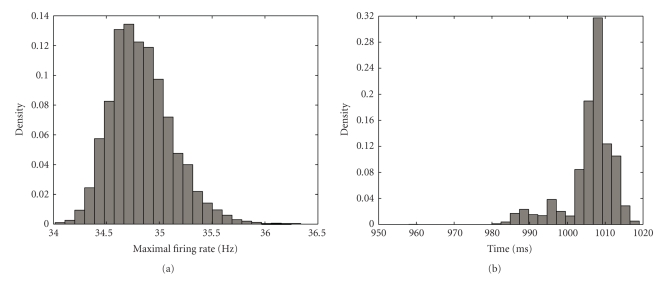
Uncertainty in the maximal firing rate for hippocampal data in Figure 4. (a) Estimated distribution of maximal firing rates computed using the algorithm in Appendix D. (b) Estimated distribution of the location in time of maximal firing.

Using a similar Monte Carlo approach (see Appendix D), it is also possible to examine in more detail the peak in firing rate that occurs at around 1000 milliseconds (Figure 7). We can compute both the distribution of maximal firing rates (Figure 7(a)) and the distribution of times that the peak is likely to occur (Figure 7(b)). We find that the 95% confidence intervals for maximal firing rate and time of occurrence (based on 10 000 Monte Carlo samples) are (34.41, 35.35) Hz and (990, 1014) milliseconds, respectively. The 95% confidence intervals provided by BARS for maximal firing rate and time of occurrence are (30.04, 36.67) Hz and (872.70, 1700) milliseconds, respectively. Thus, the state-space approach provides tighter confidence intervals than BARS for both maximal firing rate and time of occurrence. The cubic splines approach does not provide confidence bounds so comparison with this model is not possible.

**Data Example 2: estimation of the firing rate across trials in a location-scene association task**

**Figure 8:**
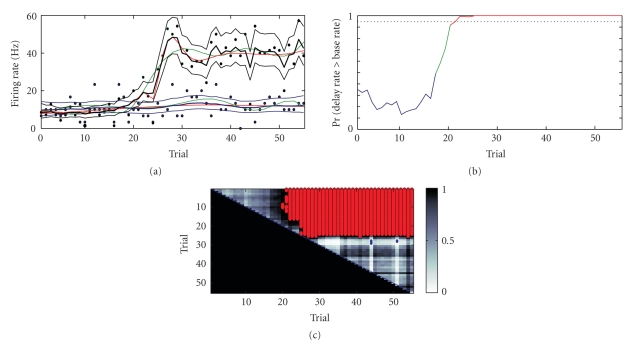
(a) Firing rates with 95% confidence limits in delay period (black) and baseline period (blue). Raw count data is shown as dots for delay (black) and baseline (blue). BARS (splines) results for delay and baseline are red (green). (b) Probability of the two firing rates estimated using the state-space method in panel A being different as a function of trial. By trial 21, the ideal observer can be confident that the firing rate in the delay period is significantly different (*P* > .95) from the firing rate in the baseline period. Line colors indicate *P* ≤ .5 (blue), .5 < *P* ≤ .95 (green), and *P* > .95 (red). (c) Trial-to-trial comparisons for the delay period firing rate showing Pr(trial *i* (*x*-axis) greater than trial *j* (*y*-axis)). The magnitude of the probabilities is represented with using the grayscale shown. Red pixels indicate where this probability is greater than 0.99. Blue pixels indicate where the probability surface falls below 0.01. From around trial 20 onwards and for the rest of the experiment, the firing rate is above the firing rate at earlier trials.

In our second example, we consider the same data as the previous example only here we are interested in tracking neural activity as a function of trial number. The neural data is divided into distinct time periods based on the timing of the stimuli shown in the trail. Each trial is initiated with the animal fixating a central fixation spot. These time periods include a baseline period (0–300 milliseconds after fixation), a scene period (301–800 milliseconds after fixation), a delay period (801–1500 milliseconds after fixation) and a response period (1501–1700 milliseconds after fixation). We seek in this example to determine the earliest trial where we can say that the firing rate during the delay period is significantly above that in the baseline period. Thus, we analyze the count data for the delay and baseline periods as a function of trial number in the session (Figure 8(a), Hz-scaled black and blue dots, resp.).

From examination of the median firing rate estimates from our state-space model, it is evident that the rate from the delay period (broad black curve, Figure 8(a)) is approximately the same as that of the firing rate of the baseline (broad blue curve) until around trials 20–25. We can formally compare the two distributions using Monte Carlo (Figure 8(b)) and find that the delay rate is significantly higher than the baseline rate from trial 20 onwards at a significance level of 0.05. As before the BARS estimate with 8 parameters and spline estimate with 27 knots (red and green curves, respectively, Figure 8(a)) are slightly smoother and lie within the 95% confidence limits estimated by the state-space approach. The cubic splines technique has difficulty tracking the rapid increase in firing rate around trial 15 (green curve, Figure 8(a)) in the delay period. Therefore, for this data our state-space results seem comparable to the results from BARS. Both models results appear preferable to the results from cubic splines, which appears to oversmooth the data.

In addition to comparing the delay rate with the baseline rate, we can also employ the algorithm in Appendix C to compare the rates between trials. We show results for the delay period (Figure 8(c)). The red block in the probability surface indicates that from around trial 20 onwards the firing rate is significantly higher than earlier trials, consistent with the baseline comparison observation.

We carried out a *χ* ^2^ goodness-of-fit test for all three methods and found that splines and BARS did not fit the data (*P* < .05), while the state-space approach did (*χ* ^2^ = 36.98, *P* = .96).

**Data Example 3: estimation of firing rate for supplemental eye field activity**

As a third example of our technique applied to real data, we consider the supplementary eye field data from Olson et al. [22] as described in Section 2. The data consists of spike counts from 60 repeated trials binned in 10-millisecond intervals over trials of length 1100 milliseconds.

**Figure 9:**
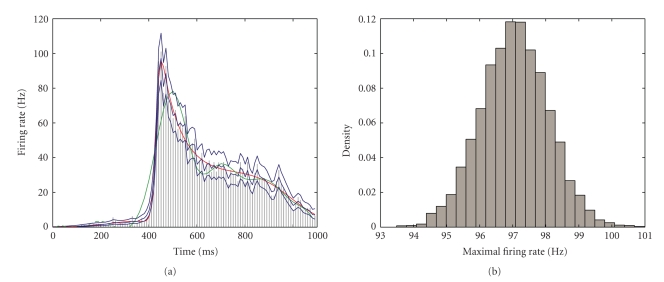
(a) PSTH of raw data (gray bars) from SEF study in Olson et al. [22]. The state-space estimates (blue lines representing median and 95% confidence bounds) track the PSTH values. Also shown are estimates by BARS (red) and splines (green). (b) The distribution of estimated values of maximal firing rate computed using the algorithm in Appendix D.

The PSTH of the raw data (gray bars, Figure 9(a)) indicates a sharp peak around 400 milliseconds. However, estimation of the position of maximal firing is difficult given the noisy nature of the PSTH. The state-space estimates of median firing rate and 95% confidence bounds (blue curves, Figure 8(a)) are also noisy reflecting the noisiness of the data. BARS (red curve) and splines (55 knots, green curve) smooth the data to a greater extent and lie largely within the 95% confidence bounds of the state-space estimates. One exception is where the splines method fails to track the rapid increases in rate around trial 400 and appears to oversmooth the data. This is also the case when the rate suddenly drops around 500 milliseconds.

The results of the chi-squared goodness-of-fit tests indicate that the state-space method (*χ* ^2^ = 39.55, *P* = 1.00) and BARS (*χ* ^2^ = 34.40, *P* = 1.00) fit the data whereas splines did not (*P* < .05). All three methods provided estimated average firing rates close to the observed firing rate of 22.85 Hz : SS (22.96 ± 22.86 Hz), BARS (22.88 ± 23.04 Hz), and splines (22.65 ± 21.88).

One important feature of this experiment was to find the location and magnitude of the maximal firing rate. To find the estimated maximal firing rate, we used Monte Carlo simulation (Appendix D) to get the distribution of the maximal firing rate and its time of occurrence (Figure 9(b)). The 95% confidence interval for maximal firing rate (based on 10 000 Monte Carlo samples) is (95.31, 98.77) Hz with time equal 450 milliseconds. The 95% confidence intervals provided by BARS for maximal firing rate and time of occurrence are (94, 102) Hz and (446, 456) milliseconds, respectively. Once again the state-space approach provided smaller confidence intervals than BARS for both maximal firing rate and time of occurrence.

## 4. Discussion

We present a state-space approach that allows the experimenter to input data at the precision of the measurements and provides a computationally efficient estimate of the firing rate and its confidence limits. The approach also allows the experimenter to investigate particular features of the firing rate curve such as when it differs significantly from baseline. It also provides confidence limits on features of interest in the firing rate such as the location and magnitude of the peak. These additional features provide a powerful set of tools with which one can analyze a wide range of neurophysiological experiments. This framework for analyzing spike train data can also be easily integrated with results from an analogous state-space model developed to analyze changes in behavioral learning [36].

### 4.1. State-Space Technique versus Other Techniques

The state-space approach compares favorably with the other two smoothing techniques considered. The confidence intervals are consistent across a range of reasonable bin width values for the PSTH (Figure 5). Thus using our state-space method, the experimenter no longer needs to run through a range of bin sizes often required when constructing a PSTH. Overall, based on MSE results for the high noise parts of the simulation study and on the chi-square results, we have found the splines' fit suboptimal. A comparison with BARS, where the spline knots' positions are chosen as part of the estimation process, indicates that our method is equally suitable for the cases considered. The fact that our computed MSEs are slightly lower than those of BARS may be due to the fact that our test function (10) was by design less smooth than the functions tested in Dimatteo et al. [21]. BARS, which uses splines, assumes that the underlying function is smooth. Because we use a first-order random walk model, our smoothness constraint is weaker. While BARS is theoretically superior for both continuous and point process observations, algorithms like BARS that rely on Monte Carlo Markov chain methods are generally more computationally intensive than our simple filter-based state-space model. BARS has recently been updated for speed and use on different computer platforms [13]: our analyses made use of an earlier C version. A typical CPU time for estimation of the state-space model for a 55 (550) trial dataset is 1.5 (5) seconds on a 2.4 Ghz computer with 2 GB RAM.

### 4.2. Choice of Initial Conditions in the State-Space Formulation

For our simulation study we considered two formulations for our state-space model. We found that using a naive estimate of initial firing rate based on a few initial data points led to a random walk model that tracked the data so well that there was practically no smoothing. This model would perform poorly in real data situations where there is noise. We modified our approach by introducing a preprocessing step. By making use of the Markov-properties of the model, we reversed the data, made a maximum likelihood estimate of our end point and then used this value as a fixed initial condition for our implementation of the model. This resulted in a smoother estimate of firing rate, more consistent with the true data in the simulation study. A similar count data model [17] has recently been implemented in a Bayesian framework [37]. In this case, what in our model appears to be sensitivity to initial conditions appears as sensitivity to choice of priors on the random walk variance in the Bayesian formulation. Congdon [37] suggests in this context that crossvalidation, by selectively omitting data and using prediction by the remaining data, may be an alternative method for choosing the correct level of smoothing.

### 4.3. Practical Applications: The Neurophysiology of Associative Learning

As illustrated in the examples taken for the location-scene association task, this state-space algorithm provides an accurate way to describe the within trial dynamics as well as the across trial dynamics illustrated in the raster plot of Figure 4(a). This state-space framework of the analysis of firing rate also provides confidence bounds as a way to measure differences in firing rate of any combination of time intervals both within a across a trial. One of the key questions we asked in this original study was when does neural activity change relative to behavioral learning. We have previously described an analogous state-space algorithm designed to provide an accurate trial by trial estimate of a subject's probability correct behavioral performance that also includes confidence bounds. Thus a trial number of learning can be defined statistically as the trial in which the lower confidence bound just passes chance performance. The development of the current state-space algorithm in the same framework as the behavioral algorithm allows us now to analyze dynamically changing behavioral and neural data from our learning experiments in the same statistical framework making comparison across the two measures much easier to interpret. These state-space approaches can be applied to a wide range of neurophysiological learning experiments across species. Importantly, the state-space algorithm for estimating spike rate functions is not limited to learning experiments but is applicable to any neurophysiological experiment in which the characterization of neural activity in response to either externally or internally driven stimuli is the goal.

### 4.4. Future Applications

In the future this model can be extended to include an arbitrary level of smoothness. This might be done by increasing the order of the autoregressive model in (2), thereby adjusting the stochastic smoothness criterion in the final (penalty) term in the likelihood (5). In general, as the order is increased the time dependence across observations increases and we might expect the rate estimates to be smoother in the case of noisy data. Selection between models can then be performed using Akaike's Information Criterion (AIC).

## Appendices

### A. Derivation of the EM Algorithm

The use of the EM algorithm to compute the maximum likelihood estimate of parameters *θ* requires us to maximize the expectation of the complete data log-likelihood. The complete data likelihood is the joint probability density of *N* and *x*, which for our model is

(A.1) $$\begin{array}{ll} & p\left(N,x\;|\;\theta \right)\\ & =\overset{J}{\underset{j=1}{\prod }}\exp\;\left[\overset{J}{\underset{j=1}{\sum }}\left[\overset{K}{\underset{k=1}{\sum }}\left(n_{jk}\log\lambda \left(k\Delta \;|\;x_{k}\right)\Delta -\lambda \left(k\Delta \;|\;x_{k}\right)\Delta \right)\right]\right]\\ & \;\times \overset{K}{\underset{k=2}{\prod }}\left({\left(2\pi \sigma_{\varepsilon }^{2}\right)}^{-1/2}\exp\;\left[{\left(-2\sigma_{\varepsilon }^{2}\right)}^{-1}{\left(x_{k}-x_{k-1}\right)}^{2}\right]\right), \end{array}$$

where the first term on the right-hand side of (A.1) is defined by the point process observation model in (2) and (3) and the second term is defined by the Gaussian probability density in (1) We compute the initial mean and variance in a preprocessing stage (see the end of this section). Assuming now that the initial mean and variance *x*_1_ and *σ*_1_^2^ are known, at iteration (*ℓ* + 1) of the algorithm we compute in the E-step the expectation of the complete data log likelihood, given the responses *N* across the *J* trials, the initial conditions, and *θ* ^(*ℓ*)^ = (*σ*_*ε*_^2^ ^(*ℓ*)^), the parameter estimate from iteration *ℓ*, which is described as follows:

E-Step

(A.2) $$\begin{array}{ll} & E\left[\log\;\left(p\left(N,x\;|\;\theta \right)\right)\;\|\;N,\theta^{\left(\ell \right)}\right]\\ & =E[\overset{J}{\underset{j=1}{\sum }}\left[\overset{K}{\underset{k=1}{\sum }}\left(n_{jk}\log\;\;\lambda \left(k\Delta \;|\;x_{k}\right)\Delta -\lambda \left(k\Delta \;|\;x_{k}\right)\Delta \right)\right]\times \;\|\;N,\theta^{\left(\ell \right)},x_{1},\sigma_{1}^{2}]\\ & \;+[-2^{-1}\left(K-2\right)\log\;\;\left(2\pi \sigma_{\varepsilon }^{2}\right)-{\left(2\sigma_{\varepsilon }^{2}\right)}^{-1}\overset{K}{\underset{k=2}{\sum }}{\left(x_{k}-x_{k-1}\right)}^{2}\;\|\;N,\theta^{\left(\ell \right)},x_{1},\sigma_{1}^{2}]. \end{array}$$

To evaluate the E-step, we have to estimate the terms

(A.3) $$\begin{array}{cc} x_{k\;|\;K} & \equiv E\left[x_{k}\;\|\;N,\theta^{\left(\ell \right)},x_{1},\sigma_{1}^{2}\right],\\ W_{k\;|\;K} & \equiv E\left[x_{x-1}\;\|\;N,\theta^{\left(\ell \right)},x_{1},\sigma_{1}^{2}\right],\\ W_{k-1,k\;|\;K} & \equiv E\left[x_{k}^{2}\;\|\;N,\theta^{\left(\ell \right)},x_{1},\sigma_{1}^{2}\right]. \end{array}$$

for *k* = 1,…, *K*, where the notation *k* ∣ *j* denotes the expectation of the state variable at *k* given the responses up to time *j*. To compute these quantities efficiently we decompose the E-step into three parts: a nonlinear recursive filter algorithm to compute *x*_*k*∣*k*_ and *W*_*k*∣*k*_, a fixed interval smoothing algorithm to estimate *x*_*k*∣*K*_ and *W*_*k*∣*K*_, and a state-space covariance algorithm to compute *W*_*k*,*k*−1∣*K*_.

#### A.1. Filter Algorithm

Given *θ* ^(*ℓ*)^ we can first compute recursively the state variable, *x*_*k*∣*k*_, and its variance, *σ*_*k*∣*k*_^2^. We accomplish this using the following nonlinear filter algorithm that is easily derived for our model in (2) to (4) using the arguments in Smith and Brown [30]:

(A.4) $$\begin{array}{c} \begin{array}{c} x_{k\;|\;k-1}=x_{k-1\;|\;k-1}, \end{array} \end{array}$$

(A.5) $$\begin{array}{c} \sigma_{k\;|\;k-1}^{2}={\sigma^{2}}_{k-1\;|\;k-1}+\sigma_{\varepsilon }^{2}, \end{array}$$

(A.6) $$\begin{array}{c} x_{k\;|\;k}=x_{k\;|\;k-1}+\sigma_{k\;|\;k-1}^{2}\left[n_{k}-\exp\;\left(x_{k|k}\right)\right], \end{array}$$

(A.7) $$\begin{array}{c} \sigma_{k|k}^{2}={\left[{\left(\sigma_{k|k-1}^{2}\right)}^{-1}+\exp\;\left(x_{k|k}\right)\right]}^{-1}, \end{array}$$

for *k* = 2,…, *K*, and the fixed initial conditions, *x*_1∣*k*_ = *x*_1_ and *σ*_1∣*k*_^2^ = *σ*_1_^2^.

Given the filter algorithm in (A.4) to (A.7), we compute *x*_*k*∣*K*_ and *W*_*k*∣*K*_ from the fixed interval smoothing algorithm in (2.17)–(2.19) of Smith and Brown [15] and we compute *W*_*k*−1∣*k*_ from the covariance smoothing algorithm using (2.20) of Smith and Brown [15]. The variance and covariance terms required for the E-step are

(A.8) $$\begin{array}{c} W_{k\;|\;K}=\sigma_{k\;|\;K}^{2}+x_{k\;|\;K}^{2},\\ W_{k-1,k\;|\;K}=\sigma_{k-1,k\;|\;K}+x_{k-1\;|\;K}x_{k\;|\;K.} \end{array}$$

In the M-step, we maximize the expected value of the complete data log likelihood in (A.2) with respect to *θ* ^(*ℓ*+1)^ giving

M-Step

(A.9) $$\sigma_{\varepsilon }^{2\left(\ell +1\right)}=[2\overset{K}{\underset{k=2}{\sum }}W_{k\;|\;K}{\left(K-2\right)}^{-1}-2\overset{K}{\underset{k=3}{\sum }}W_{k-1,k\;|\;K}^{}+x_{1\;|\;K}^{2}-2x_{1\;|\;K}^{}x_{2\;|\;K}^{}-W_{K\;|\;K}]$$

The algorithm iterates between the E-Step (A.2) and the M-Step (A.9) using the filter algorithm, the fixed interval smoothing algorithm and the state covariance algorithm to evaluate the E-step. The maximum likelihood estimate of *θ* is *θ* ^(*∞*)^. The convergence criteria for the algorithm are those used in Smith and Brown [15]. The fixed interval smoothing algorithm evaluated at maximum likelihood estimate of *θ* together with (4) give the empirical Bayes' or smoothing algorithm estimate of the spike rate function.

#### A.2. Estimation of Initial Conditions

We estimated the initial conditions *x*_1_ and *σ*_1_^2^ as part of a preprocessing stage. To do this, we reversed the temporal order of the count data *N* and applied an EM procedure as above only in this case adding a second unknown parameter to *θ*, the initial state *x*_0∣*K*_. These calculations yielded a maximum likelihood estimates of the final mean and variance of the reversed data, *x*_*K*∣*K*_ and *σ*_*K*∣*K*_^2^. We took the initial state to be normally distributed with mean *x*_1_ = *x*_*K*∣*K*_ and variance *σ*_1_^2^ = *σ*_*K*∣*K*_^2^ as our fixed initial conditions for our EM algorithm (A.1–A.9).

### B. Computing Confidence Intervals by Monte Carlo Methods

Given *ξ* ∈ (0, 1), the 1 − *ξ* confidence intervals for a given time *k*Δ can be computed from the probability density in (7) by using either Monte Carlo methods or numerical integration to compute the *ξ*/2 and the 1 − *ξ*/2 quantiles of this probability density [15]. The confidence intervals for the smoothed histogram estimate are most efficiently computed by Monte Carlo methods. To implement the algorithm we pick *I* and for *i* = 1,…, *I*, we carry out the following three steps:

(1) For *k* = 1,…, *K*, draw a realization *i* of the state process *x*_*k*∣*K*_^*i*^ using the filter algorithm (A.4)–(A.7) and the fixed interval smoothing algorithm in [15, equations (2.17)–(2.19)] evaluated at $\hat{\theta }$ the maximum likelihood estimate.

(2) For each *t*_1_ and *t*_2_, the left and right end point of a given time bin, compute ${\hat{\Lambda }}^{\left(i\right)}\left(t_{2}-t_{1}\right)=\sum_{t_{1}\leq k\Delta \leq t_{2}}\lambda \left(k\Delta \;|\;x_{k|K}^{i},\hat{\theta }\right)\Delta .$
(3) Compute the lower and upper limits of the 1 − *ξ* confidence intervals, respectively, as the *ξ*/2 and 1 − *ξ*/2 quantiles of the Monte Carlo probability density of $\hat{\Lambda }\left(t_{2}-t_{1}\right)$.

We take *I* = 10 000.

### C. Comparing the Magnitude of the Spike Rate Function at Two Different Times

To compare whether the spike rate function at one time is significantly greater than the rate function at another time, we note that the approximate posterior probability density of the state process is a *K* + 1-dimensional Gaussian probability density whose mean is defined by *x*_0∣*K*_ and *x*_*k*∣*K*_ for *k* = 1,…, *K* and whose covariance matrix is given by the fixed interval smoothing algorithm [15, equations (2.17)–(2.19)] and covariance smoothing algorithm [15, equation (2.20)]. Given times *k*Δ and *j*Δ, we wish to compute Pr(*λ*(*k*Δ ∣ *x*_*k*∣*K*_) > *λ*(*j*Δ ∣ *x*_*j*∣*K*_)). We pick *I* and proceed as follows:

(1) set *i* = 1; *S*_*I*_ = 0;
(2) draw *x*_*k*∣*K*_^*j*^ and *x*_*j*∣*K*_^*i*^ from their joint probability density;
(3) if *λ* ^*i*^(*k*Δ ∣ *x*_*k*|*K*_) > *λ* ^*i*^(*j*Δ | *x*_*j*|*K*_), then *S*_*I*_ = *S*_*I*_ + 1;
(4) *i* = *i* + 1;
(5) if *i* > *I* stop; else go to 2.

We compute Pr(*λ*(*k*Δ ∣ *x*_*k*∣*K*_) > *λ*(*j*Δ ∣ *x*_*j*∣*K*_)) ≐ *I* ^−1^ *S*_*I*_. In our analyses we chose *I* = 10 000.

### D. Computing Distributions of the Maximal Firing Rate and Their Times of Occurrences by Monte Carlo Methods

Given *ξ* ∈ (0,1), the 1 − *ξ* confidence intervals for a given time *k*Δ can be computed from the probability density in (7) by using either Monte Carlo methods or numerical integration to compute the *ξ*/2 and the 1 − *ξ*/2 quantiles of this probability density [31]. The confidence intervals of the maximal firing rate and its time of occurrence for the smoothed histogram estimate are most efficiently computed by Monte Carlo methods. To implement the algorithm we pick *J* for *j* = 1,…, *J* and we pick *I* for *i* = 1,…, *I*, and carry out the following four steps.

(1) For *k* = 1,…, *K*, draw a realization *i* of the state process *x*_*k*∣*K*_^*j*^ using the filter algorithm (A.4–A.7) and the fixed interval smoothing algorithm in [31, (2.17)–(2.19)] evaluated at $\hat{\theta }$ the maximum likelihood estimate.
(2) For each *t*_1_ and *t*_2_, the left and right end point of a given time bin, compute ${\hat{\Lambda }}^{\left(i\right)}\left(t_{2}-t_{1}\right)=\sum_{t_{1}\leq k\Delta \leq t_{2}}\lambda \left(k\Delta \;|\;x_{k|K}^{i},\hat{\theta }\right)\Delta .$
(3) Compute $\max\;\left({\hat{\Lambda }}^{\left(i\right)}\right)$ and *time* $\left(\max\;\left({\hat{\Lambda }}^{\left(i\right)}\right)\right)$.
(4) Compute $MF^{\left(j\right)}=\max\;({\hat{\Lambda }}^{\left(i\right)})$ and $MT^{\left(j\right)}=\text{time}(\max\;({\hat{\Lambda }}^{\left(i\right)}))$.
(5) Compute the lower and upper limits of the 1 − *ξ* confidence intervals, respectively, as the *ξ*/2 and 1 − *ξ*/2 quantiles of the Monte Carlo probability density of *MF* ^(*j*)^ and *MT* ^(*j*)^.

We take *J* = *I* = 10 000.
